# A Bioconductor/R Workflow for the Detection and Visualization of Differential Chromatin Loops

**DOI:** 10.12688/f1000research.153949.1

**Published:** 2024-11-11

**Authors:** JP Flores, Eric Davis, Nicole Kramer, Michael I Love, Douglas H Phanstiel

**Affiliations:** 1 Curriculum in Bioinformatics & Computational Biology, Department of Genetics, University of North Carolina at Chapel Hill, Chapel Hill, NC, 27514, USA; 2 Thurston Arthritis Research Center, University of North Carolina at Chapel Hill, Chapel Hill, NC, 27514, USA; 3 Department of Genetics, University of North Carolina at Chapel Hill, Chapel Hill, NC, 27514, USA; 4 Department of Biostatistics, University of North Carolina at Chapel Hill, Chapel Hill, NC, 27514, USA; 5 Curriculum in Genetics & Molecular Biology, Department of Genetics, University of North Carolina at Chapel Hill, Chapel Hill, NC, 27514, USA; 6 Department of Cell Biology & Physiology, University of North Carolina at Chapel Hill, Chapel Hill, North Carolina, 27514, USA; 7 Lineberger Comprehensive Cancer Center, University of North Carolina at Chapel Hill, Chapel Hill, NC, 27514, USA

**Keywords:** Hi-C, differential analysis, data visualization, 3D chromatin structure

## Abstract

**Background:**

Chromatin loops play a critical role in gene regulation by connecting regulatory loci and gene promoters. The identification of changes in chromatin looping between cell types or biological conditions is an important task for understanding gene regulation; however, the manipulation, statistical analysis, and visualization of data sets describing 3D chromatin structure is challenging due to the large and complex nature of the relevant data sets.

**Methods:**

Here, we describe a workflow for identifying and visualizing differential chromatin loops from Hi-C data from two biological conditions using the ‘mariner’, ‘DESeq2’ and ‘plotgardener’ Bioconductor/R packages. The workflow assumes that Hi-C data has been processed into ‘.hic’ or ‘.cool’ files and that loops have been identified using an existing loop-calling algorithm.

**Results:**

First, the ‘mariner’ package is used to merge redundant loop calls and extract interaction frequency counts. Next, ‘DESeq2’ is used to identify loops that exhibit differential contact frequencies between conditions. Finally, ‘plotgardener’ is used to visualize differential loops.

**Conclusion:**

Chromatin interaction data is an important modality for understanding the mechanisms of transcriptional regulation. The workflow presented here outlines the use of ‘mariner’ as a tool to manipulate, extract, and aggregate chromatin interaction data, ‘DESeq2’ to perform differential analysis of these data across conditions, samples, and replicates, and ‘plotgardener’ to explore and visualize the results.

## Introduction

3D chromatin structure plays a critical role in gene regulation, cellular differentiation, and response to external stimuli. ^1^^–^^3^ Specifically, chromatin loops connect regulatory loci, such as enhancers, to gene promoters tuning levels of transcriptional. ^4^^,^^5^ Understanding how these loops change across cell types and conditions is vital for a mechanistic understanding of transcriptional control in mammalian cells and can inform our understanding of human disease. ^6^^–^^9^ The most comprehensive views of 3D chromatin structure are provided by Hi-C ^10^^,^^11^ and Micro-C ^12^ data sets; however the large and complex nature of these data sets make them challenging to manipulate and analyze. Here, we describe a Bioconductor ^13^ workflow for the detection and visualization of differential loops from Hi-C data.

We apply this workflow to identify loops formed by an oncogenic fusion protein that is associated with acute myeloid leukemia (AML) and related hematopoietic malignancies. Most loops identified to date are driven by CTCF and cohesin proteins, which work harmoniously via loop extrusion. ^14^^,^^15^ However, we have recently shown that an oncogenic fusion protein, NUP98-HOXA9 (NHA9), forms loops via phase separation independently of canonical loop extrusion machinery and helps drive oncogenic transcription. ^6^ Using data from that manuscript, we describe a workflow for identifying loops formed by NHA9. Specifically, we compare loops from HEK293 cells that express either wild-type NHA9 (NHA9-WT) or a phase-separation-incompetent NHA9 mutant (NHA9-MT). The assumption is that the majority of loops in both cell types are driven by CTCF/cohesin and will not change between conditions; however, loops specific to the NHA9-WT expressing cells are driven by the phase separation capabilities of NHA9.

This workflow is based largely on three packages distributed as part of the R/Bioconductor initiative. R/Bioconductor is an open-source project that houses tools for the analysis of high-throughput biological data and provides an environment for these tools to be used in-concert. This workflow uses the ‘mariner’, ^16^ ‘DESeq2’ ^17^ and ‘plotgardener’ ^18^ packages and can be completed entirely within the R programming environment. The ‘mariner’ package allows users to manipulate, extract, and aggregate chromatin interaction data quickly and efficiently. ‘DESeq2’ provides statistical methods to detect biological differences in read counts observed in high-throughput sequencing experiments, with applications to RNA-seq, ChIP-seq, ATAC-seq, and Hi-C count matrices. ‘plotgardener’ is a genomic data visualization package for R that allows users to programmatically and flexibly generate publication-quality multi-panel figures incorporating both genomic and non-genomic data. We recommend using the RStudio ^19^ environment to perform these analyses because it provides an interface for smoother and more efficient coding practices, but any suitable development platform would work. The procedure described here can be applied to any comparison of Hi-C or Micro-C data from two or more cell types or conditions and provide robust detection of differential loops.

## Methods

### Requirements and assumptions

This workflow can be applied to a variety of differential looping experiments but has several requirements and assumptions. First, the workflow requires that at least two replicates were generated in order to estimate variability and enable accurate statistical inferences. Second, it requires that the raw reads (typically ‘.fastq’ format) have been processed into Hi-C contact matrices that are stored in either ‘.hic’, ‘.cool’, or ‘.mcool’ format. This is a computationally expensive process that typically requires a computational cluster and is performed outside of the R/Bioconductor environment. Several pipelines are available to perform this initial processing step. ^20^^–^^23^ Third, this workflow assumes that the pixels representing loops have been identified. Again, this is typically performed outside the R/Bioconductor environment and can be achieved via multiple existing algorithms. ^20^^,^^24^^–^^43^ Finally, because we are using ‘DESeq2’ and must identify scaling factors, this workflow assumes that the majority of loops are not changing between conditions.

### Package installation

Detailed instructions for installation of the packages used in this workflow can be found on the R/Bioconductor website but code to install the main packages used in this workflow is shown below.

## Install Packages
BiocManager::install(c("mariner",
                       "marinerData",
                       "InteractionSet",
                       "data.table",
                       "plyranges",
                       "apeglm",
                       "DESeq2",
                       "plotgardener",
                       "RColorBrewer"))



A list of all the packages loaded in this workflow is included at the end, in the Session information section. ^16^^–^^18^^,^^44^^–^^48^ Once installed, the packages are loaded with the ‘library()’ function.

## Load packages
library(mariner)
library(marinerData)
library(InteractionSet)
library(data.table)
library(plyranges)
library(DESeq2)
library(plotgardener)
library(RColorBrewer)



## Use cases

### Experimental data

The ‘.hic’ files used in this workflow were generated by Ahn et al. ^6^ and are available via GEO with accession number GSE143465. These experiments include four replicate Hi-C experiments for each of the two conditions. The raw reads were processed into 8 ‘.hic’ files using the Juicer pipeline. ^20^ The .hic files can be downloaded using the following code (note-these are large files that take a substantial length of time to download).

## Access WT Hi-C data from GEO
wt_hicFiles <-

c("https://ftp.ncbi.nlm.nih.gov/geo/samples/GSM4259nnn/GSM4259896/suppl/GSM4259896_HEK_HiC_NUP_IDR_WT_A9_1_1_inter_30.hic",

"https://ftp.ncbi.nlm.nih.gov/geo/samples/GSM4259nnn/GSM4259897/suppl/GSM4259897_HEK_HiC_NUP_IDR_WT_A9_1_2_inter_30.hic",

"https://ftp.ncbi.nlm.nih.gov/geo/samples/GSM4259nnn/GSM4259898/suppl/GSM4259898_HEK_HiC_NUP_IDR_WT_A9_2_1_inter_30.hic",

"https://ftp.ncbi.nlm.nih.gov/geo/samples/GSM4259nnn/GSM4259899/suppl/GSM4259899_HEK_HiC_NUP_IDR_WT_A9_2_2_inter_30.hic")

## Download all WT.hic files of interest using a ‘wget’ command
for (i in seq_along(wt_hicFiles)){
  system(paste0("wget -c", wt_hicFiles[i], " -P data/"))
}

## Access FS Hi-C data from GEO
fs_hicFiles <-

c("https://ftp.ncbi.nlm.nih.gov/geo/samples/GSM4259nnn/GSM4259900/suppl/GSM4259900_HEK_HiC_NUP_IDR_FS_A9_1_1_inter_30.hic",

"https://ftp.ncbi.nlm.nih.gov/geo/samples/GSM4259nnn/GSM4259901/suppl/GSM4259901_HEK_HiC_NUP_IDR_FS_A9_1_2_inter_30.hic",

"https://ftp.ncbi.nlm.nih.gov/geo/samples/GSM4259nnn/GSM4259902/suppl/GSM4259902_HEK_HiC_NUP_IDR_FS_A9_2_1_inter_30.hic",

"https://ftp.ncbi.nlm.nih.gov/geo/samples/GSM4259nnn/GSM4259903/suppl/GSM4259903_HEK_HiC_NUP_IDR_FS_A9_2_2_inter_30.hic")

## Download all FS.hic files of interest using a ‘wget’ command
for (i in seq_along(fs_hicFiles)){
  system(paste0("wget -c ", fs_hicFiles[i], " -P data/"))
}

## Create a variable for.hic file file paths
hicFiles <- list.files("data",
                        pattern = "GSM4259*",
                        full.names = T)

## replace hicFile names with shorter easier to read names
names(hicFiles) <- c("WT_rep1","WT_rep2","WT_rep3","WT_rep4", "FS_rep1","FS_rep2","FS_rep3","FS_rep4")

## Access megaMap Hi-C data from GEO
megaMap_hicFiles <-

c("https://ftp.ncbi.nlm.nih.gov/geo/series/GSE143nnn/GSE143465/suppl/GSE143465%5FHEK%5FHiC%5FNUP%5FIDR%5FFS%5FA9%5FmegaMap%5Finter%5F30.hic",

"https://ftp.ncbi.nlm.nih.gov/geo/series/GSE143nnn/GSE143465/suppl/GSE143465%5FHEK%5FHiC%5FNUP%5FIDR%5FWT%5FA9%5FmegaMap%5Finter%5F30.hic")

## Download all megaMap.hic files of interest using a ‘wget’ command
for (i in seq_along(megaMap_hicFiles)){
  system(paste0("wget -c ", megaMap_hicFiles[i], " -P data/"))
}

## Create a variable for megaMap.hic file file paths
megaMap_files <- list.files("data",
                             pattern = "*megaMap*",
                             full.names = T)

## Replace megaMap_files names with shorter easier to read names
names(megaMap_files) <- c("megaMap_FS",
                           "megaMap_WT")



The loops for this workflow were identified using the Significant Interaction Peak (SIP) ^24^ and stored in a ‘BEDPE’ file format. The loop calls were generated by first merging all four ‘.hic’ files for each condition and then running SIP on the two resulting ‘.hic’ files. We find that typically we get more accurate loop calls after merging replicates, but it is also acceptable to call loops on each of the 8 ‘.hic’ files individually. The loop calls are available via the ‘marinerData’ package and can be loaded with the following code.

## Convert loops into GInteractions objects & expand to 10kb resolution
wtLoops <- fread(marinerData::WT_5kbLoops.txt())
wtLoopsGI <- wtLoops|>
 as_ginteractions()|>
 snapToBins(binSize = 10e3)

fsLoops <- fread (marinerData::FS_5kbLoops.txt())
fsLoopsGI <- fsLoops|>
 as_ginteractions()|>
 snapToBins(binSize = 10e3)



These loop coordinates must be modified in three ways for compatibility with the rest of the workflow. First, they must be converted from ‘BEDPE’ formatted ‘.txt’ files into ‘GInteractions’ objects in R, which we can do using the ‘as_ginteractions()’ function. Second, decreasing the resolution from 5kb to 10kb leads to increased power to detect differential loops due to an increase in the counts per pixel. For this reason, we expand the resolution of our loops from 5kb to 10kb with the ‘snapToBins()’ function.

## Show summaries of wtLoopsGI
summary(wtLoopsGI)





[1] "GInteractions object of length 12095 with 9 metadata columns"





## Show summary of fsLoopsGI
summary(fsLoopsGI)





[1] "GInteractions object of length 8566 with 9 metadata columns"



Third, because the ‘.hic’ files were assembled using a human ENSEMBL genome build that lacks the “chr” prefix to chromosomes, we also remove the “chr” prefix from the merged loops using the ‘seqlevelsStyle’ function and set it to ‘“ENSEMBL”’.

## change seqlevelsStyle such that "chr" is removed from seqnames() columns
seqlevelsStyle(wtLoopsGI) <- "ENSEMBL"
seqlevelsStyle(fsLoopsGI) <- "ENSEMBL"

## Create list of loops
loopList <- list("WT" = wtLoopsGI, "FS" = fsLoopsGI)



The resulting object contain ‘GInteractions’ data as well as several columns of metadata as shown below:

## View loops
loopList





$WT
GInteractions object with 12095 interactions and 9 metadata columns:
           seqnames1                ranges1    seqnames2                 ranges2|      color APScoreAvg ProbabilityofEnrichment
              <Rle>           <IRanges>             <Rle>                <IRanges>|<character>  <numeric>              <numeric>
       [1]          9     14460000-14470000 ---          9       14760000-14770000|  0,0,0     3.27213                  0.986854
       [2]          9     89560000-89570000 ---          9       89810000-89820000|  0,0,0     2.06276                  0.952864
       [3]          9     23720000-23730000 ---          9       23780000-23790000|  0,0,0     1.95374                  0.933563
       [4]          9   128160000-128170000 ---          9     128670000-128680000|  0,0,0     4.21739                  0.991990
       [5]          9   113100000-113110000 ---          9     113380000-113390000|  0,0,0     2.52749                  0.968201
        ...         ...                      ... ...          ...                      ... .      ...         ...                       ...
   [12091]         17    16900000-16910000 ---          17       17090000-17100000|  0,0,0     3.04485                  0.968145
   [12092]         17      7250000-7260000 ---          17         7470000-7480000|  0,0,0     2.96609                  0.982038
   [12093]         17    46880000-46890000 ---          17       46950000-46960000|  0,0,0     3.60656                  0.978988
   [12094]         17     17580000-17590000 ---         17       17690000-17700000|  0,0,0     2.25041                  0.915219
   [12095]         17     47640000-47650000 ---         17       48070000-48080000|  0,0,0     3.52043                  0.980169
           RegAPScoreAvg Avg_diffMaxNeihgboor_1 Avg_diffMaxNeihgboor_2              avg        std     value
               <numeric>              <numeric>              <numeric>            <numeric>  <numeric>  <numeric>
      [1]        2.23353                1.00594               1.645508               3.43788  0.516423    4.33205
      [2]        1.32675                1.12752               1.139458               2.05279  0.586137    3.05503
      [3]        1.40833                0.62122               0.872226               2.15958  0.377306    2.71178
      [4]        2.14430                2.21301               2.601333               2.86042  1.079651    4.82754
      [5]        1.47012                1.38765               1.560463               2.21521  0.666935    3.44868
      ...            ...                    ...                    ...                         ...         ...      ...
  [12091]        1.79613               1.273048               1.572243              2.31531  0.617247   3.44691
  [12092]        1.78843               1.487051               1.750331              2.69810  0.691201   4.01992
  [12093]        2.46229               1.035204               1.397471              2.94288  0.412916   3.86306
  [12094]        1.58783               0.665246               0.802734              1.87661  0.317383   2.46794
  [12095]        2.43317               0.810456               1.486894              3.20053  0.673346   3.92093
-------
regions: 18474 ranges and 0 metadata columns
seqinfo: 23 sequences from an unspecified genome; no seqlengths

$FS
GInteractions object with 8566 interactions and 9 metadata columns:
         seqnames1             ranges1      seqnames2                   ranges2|          color APScoreAvg ProbabilityofEnrichment RegAPScoreAvg
              <Rle>           <IRanges>            <Rle>              <IRanges>|<character>  <numeric>                <numeric>      <numeric>
     [1]          9 118640000-118650000 ---            9     119330000-119340000|  0,0,0  2.61103                  0.986044           1.41438
     [2]          9   15280000-15290000 ---            9       15400000-15410000|  0,0,0  2.45301                  0.982802           1.54370
     [3]          9   90300000-90310000 ---            9       90430000-90440000|  0,0,0  2.54382                  0.944492           1.88797
     [4]          9   17810000-17820000 ---            9       18200000-18210000|  0,0,0  4.73751                  0.996093           3.17874
     [5]          9 110180000-110190000 ---            9     111520000-111530000|  0,0,0  3.40635                  0.996545           1.80221
     ...          ...                ... ...          ...                  ... .           ...        ...                       ...              ...
  [8562]         17   58800000-58810000 ---            17      59470000-59480000|  0,0,0  3.34789                  0.990769           1.90331
  [8563]         17   46880000-46890000 ---            17      46950000-46960000|  0,0,0  3.89043                  0.982768           2.73390
  [8564]         17     6810000-6820000 ---            17        7020000-7030000|  0,0,0  3.19561                  1.000000           1.83052
  [8565]         17   28700000-28710000 ---            17      29050000-29060000|  0,0,0  3.05602                  0.972453           1.58662
  [8566]         17   80630000-80640000 ---            17      80920000-80930000|  0,0,0  2.88957                  0.971356           1.37937
  Avg_diffMaxNeihgboor_1 Avg_diffMaxNeihgboor_2                     avg     std    value
            <numeric>   <numeric>   <numeric>   <numeric>   <numeric>
      [1]    1.875585    2.121637     2.60512    0.794220     4.27231
      [2]    1.491568    1.607766     2.73756    0.856876     4.06339
      [3]    0.734045    0.797816     2.23905    0.404984     2.89153
      [4]    0.971099    2.365460     4.68239    0.683678     5.54559
      [5]    3.768934    3.891115     4.49898    1.492786     7.84915
      ...         ...         ...         ...         ...         ...
   [8562]    1.968047     2.17504     2.93624    1.128197     4.68562
   [8563]    0.906871     1.43305     3.25531    0.368257     4.06142
   [8564]    0.975828     1.25194     1.73927    0.548582     2.60667
   [8565]    1.361369     2.01811     2.38214    0.691422     3.59225
   [8566]    1.910529     1.94636     1.85494    0.672978     3.55319
-------
 regions: 14370 ranges and 0 metadata columns
 seqinfo: 23 sequences from an unspecified genome; no seqlengths



### Merging loops

Since loops were called in each condition separately, the union of these two sets will include many redundant loops. Moreover, due to the imprecise nature of loop calling, some of these shared loops will be assigned to slightly different, but nearby, pixels in each data set. Testing these redundant loop calls can lead to overcounting of differential loops as well as decreased statistical power due to multiple hypothesis testing correction. To address this issue, the ‘mariner’ package can remove redundant loops and create a set of consensus loop calls by clustering and merging loops between conditions using the ‘mergePairs()’ function. This ‘mergePairs()’ function uses ‘dbscan’ to cluster loops according to a user-defined ‘radius’ that specifies the maximum distance between two pixels for them to be considered as part of the same cluster. The user can specify the exact distance as well as the method of calculating distance. In the example below, we use a radius of 10kb and as measured from the center of pixels using the Manhattan distance. Importantly, users can also provide a ‘column’ argument for how to choose which pixel to use as the consensus pixel. In this case, we choose the pixel with the highest peak analysis score (from SIP).

## Remove redundant loops (i.e. loops that are present in both WT & FS)
mergedLoops <-
 mergePairs (loopList,
             radius = 10e3,
             method = "manhattan",
             column = "APScoreAvg",
             pos = "center")



Once clustered, ‘mergePairs()’ selects a representative pixel for each cluster using either the metadata column provided or, if ‘column=NULL’, the median of modes. Before using ‘mergePairs()’, there were 20,661 loops called across conditions and after merging loops, we have 16,491.

## View number of loops after merging
summary(mergedLoops)





[1] "MergedGInteractions object of length 16491 with 9 metadata columns"



### Extracting Hi-C Counts

To determine which loops exhibit a change in contact frequency between conditions, we next need to extract the Hi-C contacts for each loop pixel across each of our replicates and conditions. This is accomplished using the ‘mariner’ function ‘pullHicPixels()’.

## Extract pixels
pixels <- pullHicPixels(
 x = mergedLoops,
 files = hicFiles,
 binSize = 10e3)

pixels





class: InteractionMatrix
dim: count matrix with 16491 interactions and 8 file(s)
metadata(3): binSize norm matrix
assays(1): counts
rownames: NULL
rowData names(9): color APScoreAvg … std value
colnames(8): WT_rep1 WT_rep2 … FS_rep3 FS_rep4
colData names(2): files fileNames
type: MergedGInteractions
regions: 23999



To view the count matrix, we can use the ‘counts()’ function. To increase our power to detect differential loops, we filter for loops that have at least 10 counts in at least 4 samples.

## Show count matrix housed within ‘pixels’
counts(pixels)





<16491 x 8> DelayedMatrix object of  type "double":
         WT_rep1  WT_rep2 WT_rep3 ... FS_rep3 FS_rep4
    [1,]      19       22      16   .      14      13
    [2,]      29       41      25   .      31      29
    [3,]      22       20      10   .      10       5
    [4,]      12       11       9   .       8      13
    [5,]      22       16      21   .       4       5
 … .  .  . .  .  .
[16487,]      42       56      55   .      48      43
[16488,]      49       52      53   .      52      90
[16489,]      54       53      59   .      41      50
[16490,]      73      103      65   .      91     113
[16491,]      26       35      31   .      28      13





## Filter out loops with low counts (at least 10 counts in at least 4 samples)
keep <- rowSums(counts (pixels) >= 10) >= 4
pixels_filt <- pixels [keep,]



### Differential loop calling

Next, we use ‘DESeq2’ to detect loop pixels that exhibit a statistically significant change in contact frequency between conditions. ‘DEseq2’ requires three pieces of information to run: 1) a count matrix where each row represents a loop and each column represents a Hi-C replicate sample, 2) a ‘data.frame’ called ‘colData’ that contains experimental and technical information about the samples (columns of the counts matrix), and 3) a design formula that describes the way the variables in colData will be used to model the counts.

We constructed our counts matrix above using the ‘pullHicPixels()’ mariner function. We construct our ‘colData’ data frame which describes each sample's genotype and assigned replicate number using base R functions.

## Construct colData
colData <- data.frame(condition = factor (rep(c("WT", "FS"), each = 4)),
                      replicate = factor (rep(1:4, 2)))

## Add rownames to colData for DESeq object
rownames(colData) <- colnames (counts (pixels_filt))

## Ensure the colnames of the count matrix is equal to the rownames of the colData
all(colnames(counts(pixels_filt)) == rownames(colData))



We build a ‘DESeq’ dataset and test for significant changes in loop pixel counts between the “WT” and “FS” conditions. The design formula specifies the major sources of variation to control for, as well as, the covariate to test for during differential loop calling. The last factor entered in the formula should be the condition of interest, as it will be the default factor that is tested by functions called later:

## Build a DESeq Dataset
dds <- DESeqDataSetFromMatrix(
 countData = counts(pixels_filt),
 colData = colData,
 design = ~ replicate + condition)

## Perform differential expression analysis based on the Negative Binomial (a.k.a. Gamma-Poisson) distribution
dds <- DESeq (dds)

dds





class: DESeqDataSet
dim: 11961 8
metadata(1): version
assays(4): counts mu H cooks
rownames: NULL
rowData names(34): baseMean baseVar … deviance maxCooks
colnames(8): WT_rep1 WT_rep2 … FS_rep3 FS_rep4
colData names(3): condition replicate sizeFactor



To generate more accurate ‘log2foldchange’ (LFC) estimates, ‘DESeq2’ and ‘apeglm’ ^46^ implement shrinkage of the LFC estimates toward zero when the information for a feature is low, e.g. when the counts are low or highly variable (high dispersion). As with the shrinkage of dispersion estimates, LFC shrinkage uses information from all features to generate more accurate estimates. Specifically, the distribution of LFC estimates for all features is used (as a prior) to shrink the LFC estimates of genes with little information or high dispersion toward LFC estimates closer to zero. We can get shrunken LFC estimates with the code shown below:

## Get shrunken results
res <- lfcShrink(dds,
                 coef = "condition_WT_vs_FS",
                 type = "apeglm")

summary(res, alpha = 0.05)





out of 11961 with nonzero total read count
adjusted p-value < 0.05
LFC > 0 (up)       : 285, 2.4%
LFC < 0 (down)     : 55, 0.46%
outliers [1]       : 0, 0%
low counts [2]     : 0, 0%
(mean count < 6)
[1] see 'cooksCutoff' argument of ?results
[2] see 'independentFiltering' argument of ?results



### Evaluating differential results

**Figure 1.**
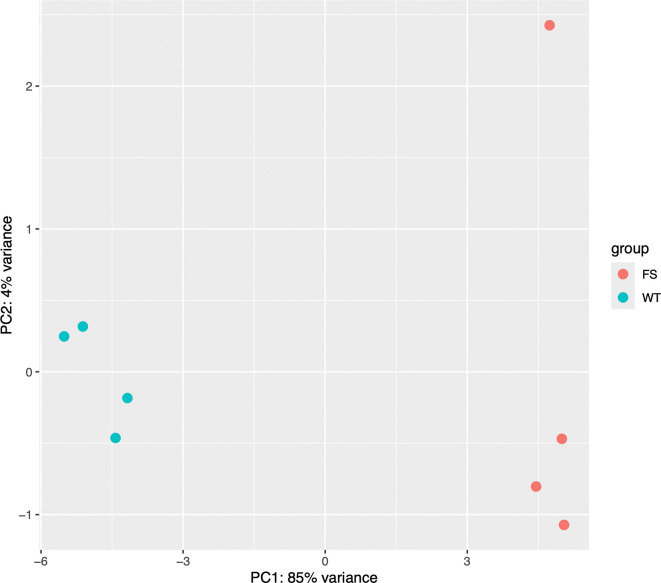
Scatter plot depicting results of PCA.

It is important to ensure that individual samples and replicates didn’t significantly skew our results. Principal Component Analysis (PCA) is a technique used to emphasize variation and bring out strong patterns in a dataset (dimensionality reduction). ‘DESeq2’ offers a simple function to generate a PCA plot from the top 500 features as shown below ( Figure 1):

## Inspect the results with a PCA plot
varianceStabilizingTransformation (dds)|>
  plotPCA(intgroup="condition") +
  ggplot2::theme(aspect.ratio = 1)



**Figure 2.**
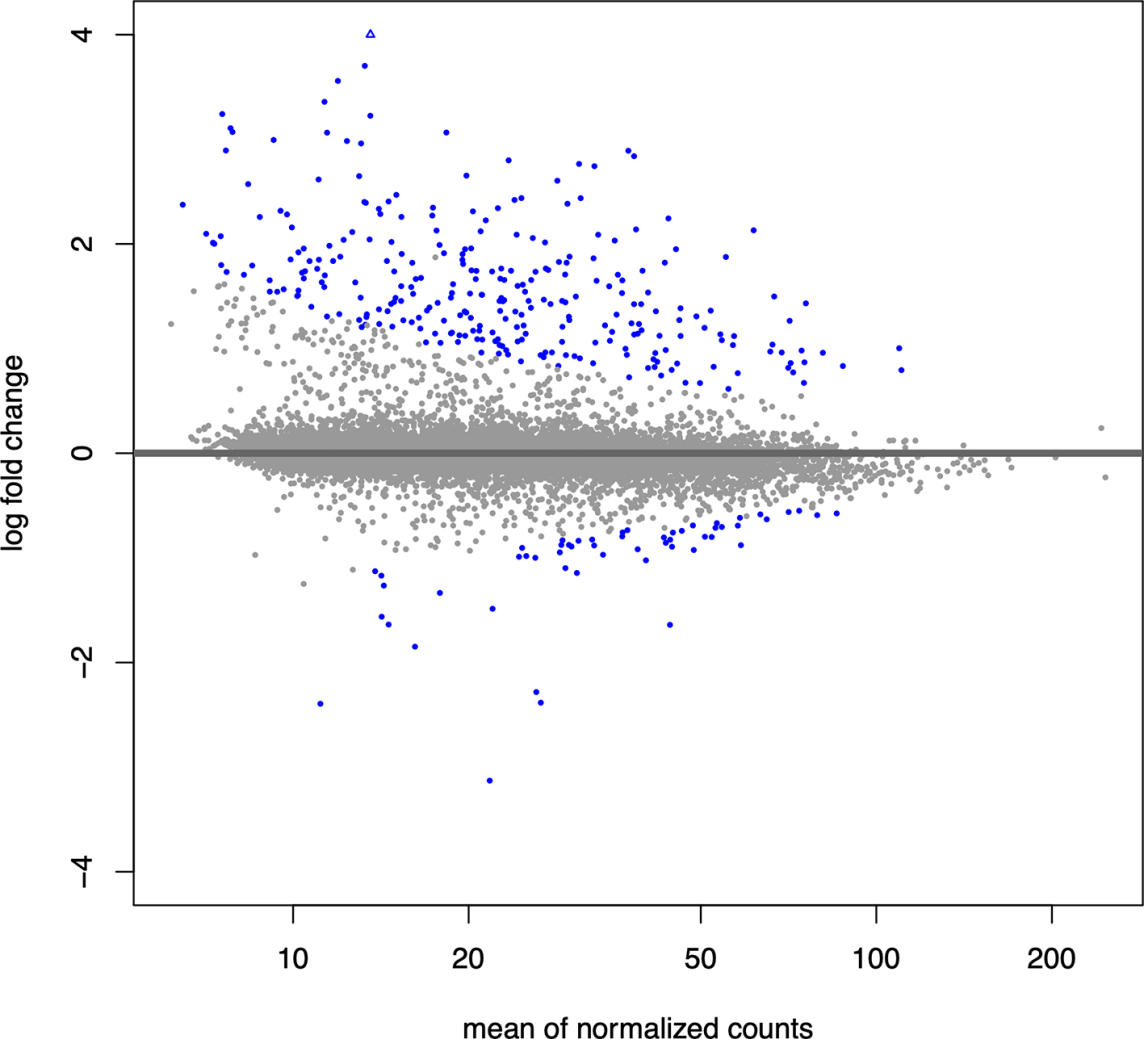
MA plot displaying normalized counts versus log2 fold change for all loops. Differential loops are shown in blue.

We next generate an MA plot to evaluate the magnitude of fold changes and how they are distributed relative to mean expression. The ‘plotMA()’ function provided by ‘DESeq2’ displays the mean of the normalized counts versus the log2 fold changes for all loops tested, and highlights differential loops in blue ( Figure 2). This allows us to assess the general distribution of effect sizes, determine their direction of effect, and provide insight into the number of differential events.

## Inspect the results with an MA plot
plotMA(res,
       alpha = 0.05,
       ylim = c(-4,4))



We can add the differential output from DESeq2 back to our ‘pixels’ object, then separate WT-specific and FS-specific loops using a BH-adjusted p-value of 0.05 and log2FoldChange above or below 0:

## Add results to rowData of our ‘pixels_filt’ InteractionMatrix
rowData(pixels_filt) <- res

## Filter for statistically significant differential loops
diffLoops <- pixels_filt[which(rowData(pixels_filt)$padj <= 0.05 &
                                 rowData(pixels_filt)$log2FoldChange > 0|
                                 rowData(pixels_filt)$log2FoldChange < 0)]



### Plot differential loops with ‘plotgardener’

Now that we have identified differential loops between “WT” and “FS” conditions, we can visualize them with ‘plotgardener’. We can visualize the differential loop with the lowest p-value using the code shown below.

First, we initialize a ‘plotgardener’ page:

## Initiate plotgardener page
pageCreate(width = 4.1, height = 4.25,
           showGuides = F)



For convenience, we make ‘pgParams’ to set parameters that are shared for all plots:

## Define shared parameters
p <- pgParams(assembly = "hg19",
              resolution = 10e3,
              chrom = "9",
              chromstart = 128420000,
              chromend = 128750000,
              zrange = c(0, 300),
              norm = "SCALE",
              x = 0.25,
              width = 3.5,
              length = 3.5,
              height = 1.5)



We plot our HiC data in rectangular format using ‘plotHicRectangle’, add loop call annotations with ‘annoPixels()’, and add a heatmap legend using ‘annoHeatmapLegend()’.

## Plot WT Hi-C Mega Map
wt_hic <- plotHicRectangle(data = megaMap_files[["megaMap_WT"]],
                           params = p,
                           y = 0.25)

## Plot FS Hi-C Map
fs_hic <- plotHicRectangle(data = megaMap_files[["megaMap_FS"]],
                           params = p,
                           y = 1.8)

## Add legend for WT Hi-C map
annoHeatmapLegend(plot = wt_hic,
                  x = 3.85,
                  y = 0.25,
                  width = 0.1,
                  height = 0.75,
                  fontcolor = 'black')

## Add legend for FS Hi-C map
annoHeatmapLegend(plot = fs_hic,
                  x = 3.85,
                  y = 1.8,
                  width = 0.1,
                  height = 0.75,
                  fontcolor = 'black')



Next, we add arrows that point to differential loops using the ‘annoPixels’ function:

## Annotate loops
annoPixels(plot = wt_hic,
           data = interactions (diffLoops),
           type = "arrow",
           shift = 2,
           col = 'black')

annoPixels(plot = fs_hic,
           data = interactions (diffLoops),
           type = "arrow",
           shift = 2,
           col = 'black')



**Figure 3.**
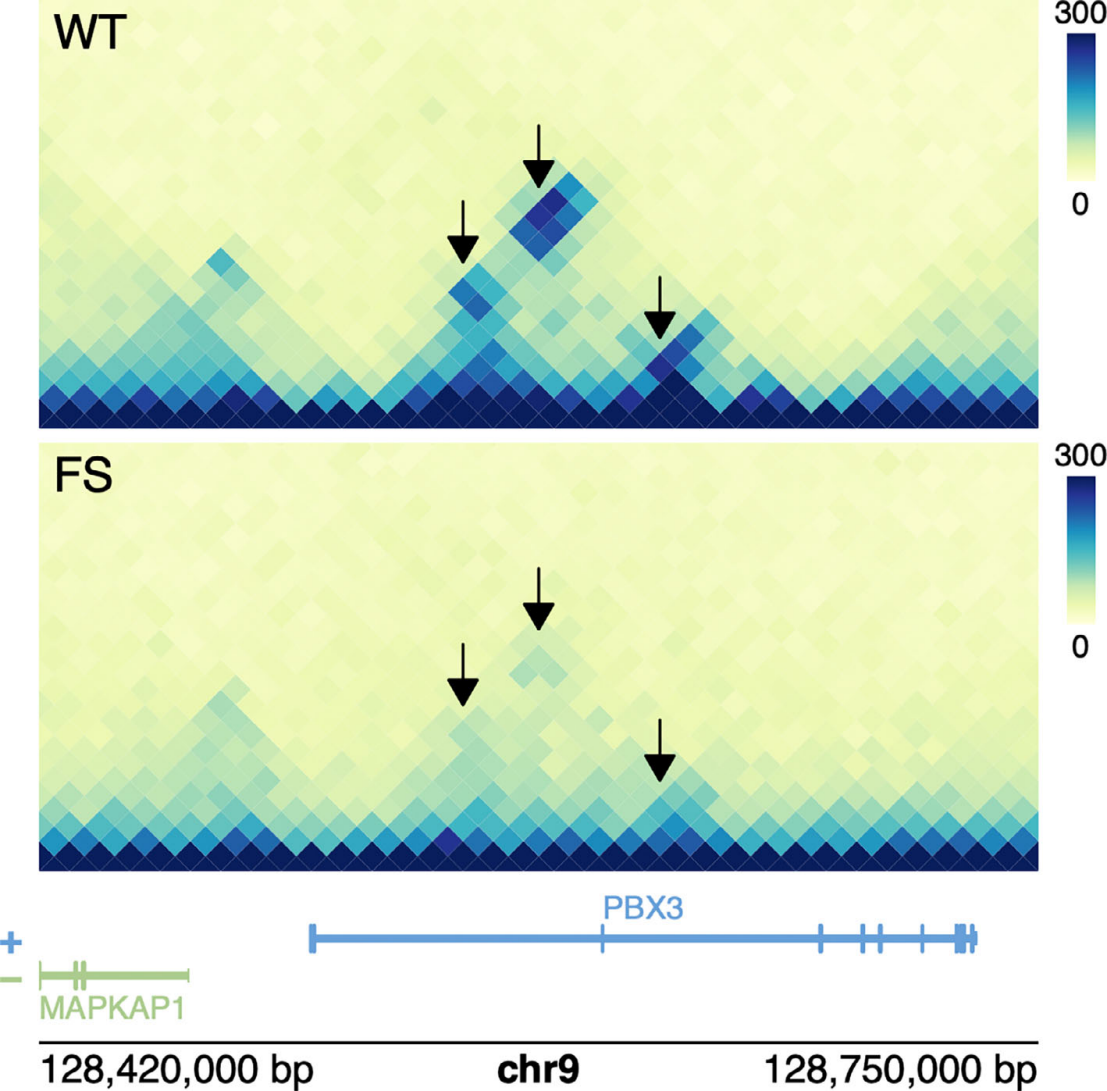
Hi-C contact maps at the PBX3 locus of cells expressing either HOXA9-NUP98-WT-IDR (top) or HOXA9-NUP98-FS-IDR. Differential loops are highlighted black.

Finally, we add text labels, a gene track for both strands, and genomic coordinates ( Figure 3):

## Add text labels
plotText(label = "WT",
         x = 0.3,
         y = 0.3,
         just = c('top', 'left'))

plotText(label = "FS",
         x = 0.3,
         y = 1.85,
         just = c('top', 'left'))

## Add Genes + Gene labels
plotGenes(chrom = paste0("chr", p$chrom),
          params = p,
          height = 0.5,
          y = 3.35)

plotGenomeLabel(params = p,
                chrom = paste0("chr", p$chrom),
                y = 3.9)



### Survey differential loops with ‘plotgardener’

An advantage to using ‘plotgardener’ instead of a genome browser is the ability to create “survey plots”. Survey plots allow us to easily generate multi-page pdfs of large sets of genomic regions. In this case, we will make a 50 page pdf displaying the Hi-C data surrounding the 50 loops with the lowest p values.

# Create Survey Plot ------------------------------------------------------

## Take Top 50 FS loops
fsLoops_50 <- head(diffLoops [order(rowData(diffLoops)$padj, decreasing = F)], 50)

## Convert to GRanges Object
fsLoops_gr <-
  GRanges(seqnames = as.character(seqnames(anchors(x = fsLoops_50, "first"))),
           ranges = IRanges(start = start(anchors(fsLoops_50, "first")),
                            end = end(anchors(fsLoops_50, "second"))),
           mcols = mcols (fsLoops_50))

## Add buffer
buffer <- 200e3
fsLoops_gr_buffer <- fsLoops_gr + buffer

## Make pdf
pdf(file = "surveyPlot.pdf",
    width = 4.1,
    height = 4.25)

## Loop through each region
for(i in seq_along(fsLoops_gr_buffer)){

   ## Initiate plotgardener page
   pageCreate(width = 4.1, height = 4.25,
              showGuides = F)

   ## Define shared parameters
   p <- pgParams(assembly = "hg19",
                 resolution = 10e3,
                 chrom = as.character(seqnames(fsLoops_gr_buffer))[i],
                 chromstart = start(fsLoops_gr_buffer)[i],
                 chromend = end(fsLoops_gr_buffer)[i],
                 zrange = c(0,300),
                 norm = "SCALE",
                 x = 0.25,
                 width = 3.5,
                 length = 3.5,
                 height = 1.5)

   ## Plot WT Hi-C Mega Map
   wt_hic <- plotHicRectangle(data = megaMap_files[["megaMap_WT"]],
                              params = p,
                              y = 0.25)

   ## Plot FS Hi-C Map
   fs_hic <- plotHicRectangle(data = megaMap_files[["megaMap_FS"]],
                              params = p,
                              y = 1.8)

   ## Add legend for WT Hi-C map
   annoHeatmapLegend(plot = wt_hic,
                     x = 3.85,
                     y = 0.25,
                     width = 0.1,
                     height = 0.75,
                     fontcolor = 'black')

   ## Add legend for FS Hi-C map
   annoHeatmapLegend(plot = fs_hic,
                     x = 3.85,
                     y = 1.8,
                     width = 0.1,
                     height = 0.75,
                     fontcolor = 'black')

   ## Annotate loops
   annoPixels(plot = wt_hic,
              data = interactions(diffLoops),
              type = "arrow",shift = 2,
              col = 'black')

  annoPixels(plot = fs_hic,
             data = interactions(diffLoops),
             type = "arrow",shift = 2,
             col = 'black')

   ## Add text labels
   plotText(label = "WT",
            x = 0.3,
            y = 0.3,
            just = c('top', 'left'))

   plotText(label = "FS",
            x = 0.3,
            y = 1.85,
            just = c('top', 'left'))

   ## Add Genes + Gene labels
   plotGenes(chrom = paste0("chr", p$chrom),
             params = p,
             height = 0.5,
             y = 3.35)

   plotGenomeLabel(params = p,
                   chrom = paste0("chr", p$chrom),
                   y = 3.9)
}
dev.off()



## Conclusion

Chromatin interaction data is an important modality for understanding the mechanisms of transcriptional regulation. The workflow presented here outlines the use of ‘mariner’ as a tool to manipulate, extract, and aggregate chromatin interaction data, ‘DESeq2’ to perform differential analysis of these data across conditions, samples, and replicates, and ‘plotgardener’ to explore and visualize the results.

Importantly, these tools work in concert within the R/Bioconductor environment, allowing for this workflow to be modular and compatible with other R/Bioconductor packages. For example, alternative Bioconductor packages for differential testing or for visualization of genomic data could easily be used within the R/Bioconductor environment. Our workflow allows for flexible input of Hi-C and Micro-C data in the form of ‘.hic’, ‘.cool’, and ‘.mcool’ files and visualizations are amenable to customizations with ‘grid’ graphics. This workflow provides a user-friendly pipeline for new and experienced genomicists and bioinformaticians.

## Packages used

Here, we report the software versions that were used to produce this workflow. To accomplish this, we run ‘sessionInfo()’:

## Get session information
sessionInfo()


R version 4.3.1 (2023-06-16)
Platform: x86_64-pc-linux-gnu (64-bit)
Running under: Red Hat Enterprise Linux 8.9 (Ootpa)

Matrix products: default
BLAS/LAPACK: /nas/longleaf/rhel8/apps/r/4.3.1/lib/libopenblas_zenp-r0.3.23.so; LAPACK version 3.11.0

locale:
 [1] LC_CTYPE=en_US.UTF-8       LC_NUMERIC=C               LC_TIME=en_US.UTF-8 LC_COLLATE=en_US.UTF-8
 [5] LC_MONETARY=en_US.UTF-8    LC_MESSAGES=en_US.UTF-8    LC_PAPER=en_US.UTF-8 LC_NAME=C
 [9] LC_ADDRESS=C               LC_TELEPHONE=C                      LC_MEASUREMENT=en_US.UTF-8      LC_IDENTIFICATION=C

time zone: America/New_York
tzcode source: system (glibc)

attached base packages:
[1] stats4   stats graphics   grDevices utils   datasets   methods base

other attached packages:
 [1] RColorBrewer_1.1-3       plotgardener_1.8.3     DESeq2_1.42.1       plyranges_1.22.0
 [5] data.table_1.15.4        InteractionSet_1.30.0  SummarizedExperiment_1.32.0 Biobase_2.62.0
 [9] MatrixGenerics_1.14.0    matrixStats_1.3.0      GenomicRanges_1.54.1  GenomeInfoDb_1.38.8
[13] IRanges_2.36.0           S4Vectors_0.40.2       BiocGenerics_0.48.1  marinerData_1.2.0
[17] mariner_1.2.1

loaded via a namespace (and not attached):
 [1] DBI_1.2.3                           bitops_1.0-7                                biomaRt_2.58.2
 [4] rlang_1.1.4                         magrittr_2.0.3                              compiler_4.3.1
 [7] RSQLite_2.3.7                       GenomicFeatures_1.54.4                      png_0.1-8
 [10] vctrs_0.6.5                        stringr_1.5.1                               pkgconfig_2.0.3
 [13] crayon_1.5.3                       fastmap_1.2.0                               dbplyr_2.5.0
 [16] XVector_0.42.0                     labeling_0.4.3                              utf8_1.2.4
 [19] promises_1.2.1                     Rsamtools_2.18.0                            strawr_0.0.92
 [22] purrr_1.0.2                        bit_4.0.5                                   zlibbioc_1.48.2
 [25] cachem_1.1.0                       progress_1.2.3                              blob_1.2.4
 [28] later_1.3.2                        rhdf5filters_1.14.1                         DelayedArray_0.28.0
 [31] interactiveDisplayBase_1.40.0      Rhdf5lib_1.24.2                             BiocParallel_1.36.0
 [34] parallel_4.3.1                     prettyunits_1.2.0                           R6_2.5.1
 [37] stringi_1.8.4                      rtracklayer_1.62.0                          Rcpp_1.0.13
 [40] assertthat_0.2.1                   httpuv_1.6.15                               Matrix_1.6-3
 [43] tidyselect_1.2.1                   rstudioapi_0.16.0                           abind_1.4-5
 [46] yaml_2.3.10                        codetools_0.2-19                            curl_5.2.1
 [49] lattice_0.22-5                     tibble_3.2.1                                KEGGREST_1.42.0
 [52] shiny_1.9.1                        withr_3.0.1                                 gridGraphics_0.5-1
 [55] BiocFileCache_2.10.2               xml2_1.3.5                                  ExperimentHub_2.10.0
 [58] Biostrings_2.70.3                  filelock_1.0.3                              pillar_1.9.0
 [61] BiocManager_1.30.22                TxDb.Hsapiens.UCSC.hg19.knownGene_3.2.2     generics_0.1.3
 [64] dbscan_1.2-0                       RCurl_1.98-1.16                             BiocVersion_3.18.1
 [67] hms_1.1.3                          ggplot2_3.5.1                               munsell_0.5.1
 [70] scales_1.3.0                       xtable_1.8-4                                glue_1.7.0
 [73] tools_4.3.1                        AnnotationHub_3.10.1                        BiocIO_1.12.0
 [76] locfit_1.5-9.8                     GenomicAlignments_1.38.2                    fs_1.6.4
 [79] XML_3.99-0.17                      rhdf5_2.46.1                                grid_4.3.1
 [82] AnnotationDbi_1.64.1               colorspace_2.1-0                            GenomeInfoDbData_1.2.11
 [85] HDF5Array_1.30.1                   restfulr_0.0.15                             cli_3.6.3
 [88] rappdirs_0.3.3                     fansi_1.0.6                                 S4Arrays_1.2.1
 [91] dplyr_1.1.4                        gtable_0.3.5                                yulab.utils_0.1.5
 [94] digest_0.6.36                      SparseArray_1.2.4                           ggplotify_0.1.2
 [97] org. Hs.eg.db_3.17.0               farver_2.1.2                                rjson_0.2.21
[100] htmltools_0.5.8.1                  memoise_2.0.1                               lifecycle_1.0.4
[103] httr_1.4.7                         colourvalues_0.3.9                          mime_0.12
[106] bit64_4.0.5



## Ethics and consent

Ethical approval and consent were not required.

## Data Availability

Gene Expression Omnibus (GEO): The Hi-C data used in this study. Accession number GSE143465; https://www.ncbi.nlm.nih.gov/geo/query/acc.cgi?acc=GSE143465. ^6^ The chromatin loop data is available as part the ‘marinerData’ package. The source code the multipage pdf file generated by this workflow are available on GitHub: https://github.com/jpflores-13/F1000R and Archived source code: Zenodo: https://doi.org/10.5281/zenodo.13899138 License for Github(‘plotgardener’ & ‘mariner’): MIT License License for Zenodo: Creative Commons Attribution 4.0 International License for ‘DESeq2’: LGPL (≥ 3) All the packages used in this workflow are publicly available from the Bioconductor project (version 3.18) and the Comprehensive R Archive Network ( CRAN).
