# Supplementary material for: A Bioconductor/R Workflow for the Detection and Visualization of Differential Chromatin Loops
Source: F1000Res. 2024 Nov 11;13:1346. [Version 1] doi: 10.12688/f1000research.153949.1 (PMC11809633; doi:10.12688/f1000research.153949.1)
Supplement: Peer review history

# Reviewer report: Reviewer response for version 1

**DOI:** 10.5256/f1000research.168915.r346529

**Published:** 2024-12-31

**Authors:** Frank Johannes, Luca Schlegel

**Affiliations:** 1 Technical University of Munich, Munich, Germany; 2 Technical University of Munich, Germany, Germany

The authors present an R-based pipeline to visualize (differential) chromatin loops using processed .hic (or .cool/.mcool) files. The final output consists of heatmap-like plots comparing chromatin loops between two conditions (e.g., wild-type NHA9 (WT) and mutant NHA9 (MT)) within a defined genomic range. The pipeline can be applied to other comparisons with appropriate Hi-C data. While the pipeline is well structured, modular and with clear data visualization, a few things can be improved:

**Major Points:**

1. Lack of Biological Motivation. The authors mention that "chromatin loops are crucial for gene regulation," but this statement lacks sufficient biological depth. Please consider expanding this section, beside your specific example of NHA9. 
2. Need for a Workflow Figure. While the paper describes the workflow in text, a small schematic figure illustrating the pipeline would capture more readers attention. This figure could include the inputs (e.g., .hic files), processing steps (e.g., loop extraction, differential analysis), and outputs (e.g., heatmaps, survey plots).
3. Lack of Comparison with Other Visualization Tools. It is not clear why one should use this R-based pipeline over other existing visualization tools like genome browsers or Hi-C heatmap generators like Juicebox and HiGlass. While the survey plot feature is highlighted as an advantage, it seems primarily focused on saving time. Are there additional benefits? A small comparison with other existing loop visualization methods would be beneficial. 

**Minor Edits:**

1. Critical Typo. The first command wget -c is missing a space after -c. Without this, the downloads fail. 
2. Unspecified Dependency. It was necessary to load the library(XML) to run the pipeline successfully. Please verify and include this dependency if required.
3. Clarify Dataset Labels. The manuscript contrasts wild-type NHA9 (WT) with a mutant (MT), but the meaning of "FS" in the filenames is not explained.

Is the rationale for developing the new method (or application) clearly explained?

Partly

Is the description of the method technically sound?

Yes

Are the conclusions about the method and its performance adequately supported by the findings presented in the article?

Yes

If any results are presented, are all the source data underlying the results available to ensure full reproducibility?

Yes

Are sufficient details provided to allow replication of the method development and its use by others?

Yes

Reviewer Expertise:

epigenomics

We confirm that we have read this submission and believe that we have an appropriate level of expertise to confirm that it is of an acceptable scientific standard, however we have significant reservations, as outlined above.

# Reviewer report: Reviewer response for version 1

**DOI:** 10.5256/f1000research.168915.r346522

**Published:** 2024-12-24

**Authors:** Raúl de Haro Blázquez

**Affiliations:** 1 Josep Carreras Leukaemia Research Institute, Barcelona, Spain

**Summary of the article:**

 The authors present a workflow that integrates three distinct packages to handle interaction data, perform loop differential analysis, and visualize the results. They provide an example using data from a previous study to demonstrate the workflow's application.

**Is the rationale for developing the new method (or application) clearly explained?**

 Yes, the authors clearly articulate the need for a well-structured combination of packages to address downstream analysis for Hi-C data.

**Is the description of the method technically sound?**

 Yes, the authors provide a detailed explanation of the three different packages used in their workflow.

**Are sufficient details provided to allow replication of the method development and its use by others?**

 Yes, the article offers comprehensive information to enable others to replicate the method.

**If any results are presented, are all the source data underlying the results available to ensure full reproducibility?**

 Yes, the data and plots are accessible on GitHub and can be easily reproduced by following the code provided in the article.

**Are the conclusions about the method and its performance adequately supported by the findings presented in the article?**

 Partly. The Conclusions section reiterates the individual conclusions of each package which are commented in the “Results” section . As the authors are presenting a workflow, a "tool" for manipulating interaction data, they should include a global conclusion about the workflow itself, highlighting the advantages of combining these specific three packages over using alternatives.

**Minor Comments**

**Introduction:**

 For a broader audience, it would be beneficial to provide more information about the biology behind the formation of the oncogenic fusion protein. The importance of selecting this specific protein becomes clearer upon consulting reference (6).

**Methods:**

 The authors mention that "this workflow assumes that the majority of loops are not changing between conditions." How does this apply to situations where we need to compare two different cell types (e.g., two related cell types in a differentiation process) without information on the extent of shared interactions? They should address this scenario or offer an alternative differential analysis method.

**Use Cases:**

 - Experimental Data: It would be helpful if the authors provided mock Hi-C files for easy download, allowing users to replicate the workflow more conveniently.

 - Extracting Hi-C Counts: The authors suggest filtering out data with fewer than 10 counts. They should explain why they chose a threshold of 10 counts. Is this based on data distribution? If so, they should mention it.

Is the rationale for developing the new method (or application) clearly explained?

Yes

Is the description of the method technically sound?

Yes

Are the conclusions about the method and its performance adequately supported by the findings presented in the article?

Partly

If any results are presented, are all the source data underlying the results available to ensure full reproducibility?

Yes

Are sufficient details provided to allow replication of the method development and its use by others?

Yes

Reviewer Expertise:

Genomics - Epigenomics - Bioinformatics

I confirm that I have read this submission and believe that I have an appropriate level of expertise to confirm that it is of an acceptable scientific standard.

# Reviewer report: Reviewer response for version 1

**DOI:** 10.5256/f1000research.168915.r346527

**Published:** 2024-12-23

**Authors:** Vanessa Vermeirssen

**Affiliations:** 1 Ghent University, Ghent, Belgium

This paper describes a workflow for identifying and visualizing differential chromatin loops from Hi-C data from two biological conditions using the ‘mariner’, ‘DESeq2’ and ‘plotgardener’ Bioconductor/R packages.

 It is technically sound and clearly described. The only comment I have is to add more details to “Evaluating differential results”: When do you conclude from the PCA that samples/replicates are skewing the results? Is top 500 features enough to plot? Also provide a result interpretation sentence for Figure 2.

Is the rationale for developing the new method (or application) clearly explained?

Yes

Is the description of the method technically sound?

Yes

Are the conclusions about the method and its performance adequately supported by the findings presented in the article?

Yes

If any results are presented, are all the source data underlying the results available to ensure full reproducibility?

Yes

Are sufficient details provided to allow replication of the method development and its use by others?

Yes

Reviewer Expertise:

Computational biology

I confirm that I have read this submission and believe that I have an appropriate level of expertise to confirm that it is of an acceptable scientific standard.

# Reviewer report: Reviewer response for version 1

**DOI:** 10.5256/f1000research.168915.r343622

**Published:** 2024-12-14

**Authors:** Benjamin Soibam

**Affiliations:** 1 University of Houston- Downtown, Houston, TX, USA

The authors provide a workflow/tool to identify and visualize differential chromatin loops from Hi-C data from two biological conditions. The tool uses existing packages in R such as the ‘mariner’, ‘DESeq2’ and ‘plotgardener’.

 3D chromatin data has been widely used to understand infer genome function. The authors have provided a workflow to compare 3D genome organization data from two conditions. This can help identify differences between the two conditions at the chromatin organization level. This tool will be very useful for many who are interested in understanding the rules govern 3D genome function and and its role in cellular processes.

Is the rationale for developing the new method (or application) clearly explained?

Yes

Is the description of the method technically sound?

Yes

Are the conclusions about the method and its performance adequately supported by the findings presented in the article?

Yes

If any results are presented, are all the source data underlying the results available to ensure full reproducibility?

Yes

Are sufficient details provided to allow replication of the method development and its use by others?

Yes

Reviewer Expertise:

Computational genomics, Biological Data Science, Applied Machine learning to understand 3D genome organization.

I confirm that I have read this submission and believe that I have an appropriate level of expertise to confirm that it is of an acceptable scientific standard.
